# Supporting families and carers during the inpatient admission

**DOI:** 10.1186/2050-2974-1-S1-O13

**Published:** 2013-11-14

**Authors:** Michelle Roberts, Michelle Hamlin

**Affiliations:** 1 Royal Brisbane and Women's Hospital, Australia

## 

The Royal Brisbane and Women's Hospital Eating Disorder Unit is a Room: State wide public specialist service providing high quality support and intervention to adults requiring an inpatient admission to treat eating disorders.

Family members and carers play a significant role in providing support for their loved one both in the community and during inpatient admissions. A gap in service delivery was noted in the provision of support to families and carers during the inpatient admission. The identification of a nominated family worker (provided by the Social Worker) was developed to ensure that families and carers were able to access support and assistance in a timely manner.

In conjunction with this an information booklet was produced to be given to families on admission of their loved ones as a way of providing detailed, up to date information to better assist and improve the support family members received.

The information booklet was developed in conjunction with consumers, a paid carer representative and other eating disorder professionals.

This abstract was presented in the **Care in Inpatient and Community Settings** stream of the 2013 ANZAED Conference.

